# Interaction Effects of Nitrogen Source and Irrigation Regime on Tuber Quality, Yield, and Water Use Efficiency of *Solanum tuberosum* L.

**DOI:** 10.3390/plants9010110

**Published:** 2020-01-15

**Authors:** Mohamed A. M. Eid, Ali A. Abdel-Salam, Haythum M. Salem, Samira E. Mahrous, Mahmoud F. Seleiman, Abdullah A. Alsadon, Talaat H. I. Solieman, Abdullah A. Ibrahim

**Affiliations:** 1 Soil, Water and Environment Research Institute (SWERI), Agricultural Research Center (ARC), P.O Box 12112 Giza, Egypt; m.eid86@yahoo.com (M.A.M.E.); samira_mahrous@yahoo.com (S.E.M.); 2 Department of Soils and Water, Faculty of Agriculture, Benha University, P.O. Box 13637 Benha, Egypt; alyabsalam@yahoo.com (A.A.A.-S.); salemh2000eg@yahoo.com (H.M.S.); 3 Plant Production Department, College of Food and Agriculture Sciences, King Saud University, P.O. Box 2460, Riyadh 11451, Saudi Arabia; alsadon@ksu.edu.sa (A.A.A.); talaat.solieman@yahoo.com (T.H.I.S.); adrahim@ksu.edu.sa (A.A.I.); 4 Department of Crop Sciences, Faculty of Agriculture, Menoufia University, Shibin El-kom 32514, Egypt; 5 Vegetable Crops Department, Faculty of Agriculture, Alexandria University, P.O. Box 21527 Alexandria, Egypt

**Keywords:** tuber yield, quality, WUE, nitrogen source, drip irrigation

## Abstract

Two field experiments were conducted to investigate the effects of three drip irrigation regimes (G_1_: 120% crop evapotranspiration (ETc), G_2_: 100% ETc, and G_3_: 80% ETc) and four nitrogen (N) source treatments (S_0_: non-fertilized; S_1_: urea, S_2_: ammonium nitrate, and S_3_: ammonium sulfate on water consumption use, water utilization efficiency, chlorophyll, yield and tubers quality of potato (*Solanum tuberosum* L.; cv Diamond) under a drip irrigation system during two successive winter seasons (2015/16 and 2016/17)). Nitrogen fertilization was applied at 380 kg ha^−1^ as standard application for potato in the investigated area. The highest tubers yield was obtained from potato grown with G_1_ S_2_ (65.8 Mg ha^−1^), G_1_ S_3_ (63.6 Mg ha^−1^), G_2_ S_2_ (64.1 Mg ha^−1^), and G_2_ S_3_ (62.4 Mg ha^−1^), while the lowest tubers yield was obtained from potato grown with G_3_ S_0_ (10.1 Mg ha^−1^) and G_2_S_0_ (17.4 Mg ha^−1^). Different treatments of N source resulted in a significant increase for water use efficiency (WU_t_E) compared with unfertilized treatment. For the interaction effect, the highest WU_t_E was obtained from potato grown with G_3_ S_2_ (18.1 kg m^−3^), followed by G_3_ S_3_ (17.6 kg m^−3^), while the lowest WU_t_E was obtained from plants grown with G_3_S_0_ (3.0 kg m^−3^). However, the highest chlorophyll content was obtained from plants grown with G1 and any N source, followed by G_2_S_1-3_, while the lowest chlorophyll content was obtained from those grown with G_3_S_0_. The highest N, S, protein, and P contents in tubers were obtained from plants grown with G_3_S_3,_ G_3_S_2_, and G_2_S_2_, while the highest K content in tubers was obtained from plants grown with G_1_S_1_ and G_1_S_2_. In concussion, the integrative effects of G_1_ or G_2_ with S_2_ or S_3_ is recommended for high productivity, while the integrative effects of G_3_S_3_ and G_3_S_2_ are recommended for high quality tubers.

## 1. Introduction

Potato (*Solanum tuberosum* L.) is a principal food crop, and an essential source of nutrients for human populations [1]. The annual world production of potato tubers, obtained from the cultivated area of 19.3 M ha during 2018, was 388.1 Tg [1]. Potatoes are widely cultivated in more than 164 countries and are consumed in fresh or processed form almost daily by more than a billion people. Potato is ranked as the fourth crop after wheat (*Triticum aestivum* L.), rice (*Oryza sativa* L.), and maize (*Zea mays* L.), among other crops according to the total production [1], and is the number one among non-grain food commodity [2]. It contains different essential dietary constituents such as essential nutrients, protein, minerals, carbohydrates, and vitamins [3]. Moreover, potatoes are not only an important food crop, as is the case with most other crops [1], but are also increasingly serving as feedstock for different industrial products [4].

The excessive inputs in agricultural systems that can achieve high productivity and quality of crops to feed a growing population are considered to be some of the most troublesome agricultural practices for environmental resources [5,6,7]. Thus, increasing food crop cultivation requires the rationalization of the inputs such as water and fertilizer applications. Additionally, due to the expense of water and its limited supply in semi-arid regions, it is an important to provide better irrigation management that can increase its effective and efficient use in order to save water [8]. Drip irrigation provides many agronomic and water conservation benefits for irrigated agriculture, such as the increased efficiency of plant nutrition [7]. There is a positive relationship between the total applied water and tuber yield, particularly under drip irrigation [7]. Agriculture is considered to be the major water user; thus, its effective use is required to preserve such limited resources. With the purpose of sustaining agricultural production, different rational agricultural water use is obligatory, especially in regions where the present irrigation practices and systems are not efficient. Therefore, irrigation management is considered to be an attractive opportunity to alleviate water shortage and scarcity in semi-arid regions [8]. Potatoes have a shallow root system, with 85% growing within the upper 40 cm of soil (in some cases, it may extend to 100 cm or more) [9]. Therefore, it is important to plan and execute an efficient irrigation schedule for potato crops. Water stress, as well as excessive water, can decrease potato yield: The former decreases plant growth and the later hinders normal plant physiological processes, since different growth stages of potato plants are sensitive to inadequate irrigation [10]. A study on potatoes conducted in semi-arid land by Fabeiro et al. [11] showed that irrigation with 5000–6500 m^3^ ha^−1^ resulted in the highest tubers yield, while using 3000 m^3^ ha^−1^ resulted in a 50% reduction in the tubers yield.

Potato yield and fertilizer applications are significantly correlated, and suitable fertilizers can significantly improve potato quality and yield [12]. Potato crops require high rates of fertilizers, particularly those containing N and K; the requirement for N is approximately twice that of K [13]. Balanced application of N and irrigation water is required to ensure high crop yields. In addition, investigating the suitable N source for high yielding, based on the soil type and climatic conditions, is one of the most important aspects. Khan et al. [14] used urea fertilization at 200 and 300 kg N ha^−1^ and obtained tuber yields of 17.4 and 18.9 Mg ha^−1^, respectively. However, the effect of ammonium sulfate, urea, and ammonium nitrate application at four rates (from 312 to 672 kg N ha^−1^) on the productivity of potatoes was investigated by Ahmed et al. [15]. They reported that the rates above 552 kg N ha^−1^ had no significant effects on tuber yield. In addition, they [15] reported that ammonium nitrate was the most efficient N source, while ammonium sulfate was the lowest efficient N source for plant growth and tuber yield. However, Khalil [16] reported that ammonium nitrate and ammonium sulfate resulted in highest tuber yields of 28.8 and 27.4 Mg ha^−1^, respectively. Ammonium sulfate can potentially provide the benefits of ammonium nitrate (i.e., steady N supply and reduced ammonia volatilization) as well as providing sulfur, since ammonium nitrate becomes less readily available in different regions. Potato fertilized with NO_3_^−^-N presented healthy shoot growth, which led to efficient tuber initiation; however, plants preferred NH_4_^+^-N after tuber initiation [17]. Vaezzadeh and Naderidarbaghshahi [18] reported no significant increase in the tuber yield at rates above 525 kg ha^−1^. Abu-Zinada [19] and Badr et al. [20] obtained higher yields when a high dose of N was applied. Alva et al. [21] stated that the application of N in the form of urea at a rate of 336 kg N ha^−1^ achieved the highest yield with high quality tubers. The results obtained from different investigations on different N source or rates differ, as mentioned above.

Among the different environmental factors, water supply and its management and nitrogen fertilization are considered to be the two most major limiting factors that affect the yield and quality of potatoes. Therefore, the objectives of the current investigation were to study the integrative effects of four N source (S0 = non-fertilized plants; S1 = urea; S2 = ammonium nitrate, and S3 = ammonium sulfate) and three regimes of the drip irrigation system (G_1_ = 120% ETc; G_2_ = 100% ETc; and G_3_ = 80% ETc) on chlorophyll, tuber and foliage yields, water use efficiency, and the quality of potato grown on a clay loam soil.

## 2. Materials and Methods

### 2.1. Site, Plant Material and Experimental Design

Two field experiments were performed at El-Qanater Horticultural Research Station, Kalubiya Governorate, Egypt (Latitude: 30°08′ N, Longitude: 31°15′ E, Elevation: 16.9 m above mean sea level) to investigate the integrative effects of three water regimes of drip irrigation and four treatments of N source on growth, WUtE, yield, and the quality of potato during two successive winter seasons (2015/2016 and 2016/2017).

The experimental design was a split-plot in a randomized complete block design with three replicates. Different drip irrigation regimes (G_1_ = irrigation at 120% crop evapotranspiration (ETc); G_2_ = 100% ETc and G_3_ = 80% ETc) were assigned into main plots. Different sources of N treatments (S_0_ = non-fertilized; S_1_ = urea; S_2_ = ammonium nitrate; and S_3_ = ammonium sulfate) were placed in sub-plots. Urea, ammonium nitrate, and ammonium sulfate contained 46%, 33%, and 21% N, respectively. The total number of treatments in the current investigation was 12 different combinations involving three irrigation regimes of drip irrigation and four treatments of N source. Nitrogen application from different source was applied on the basis of the standard and recommended rate by the Egyptian Ministry of Agriculture in the investigated region (380 kg N ha^−1^). Potatoes were cultivated on the fifth and third of October in the first and second season, respectively. The diameter of the used tubers in the cultivation process was 35–45 mm, and the seeding rate was 2.7 Mg ha^−1^. The sub-plot area was 20 m^2^ (4 × 5 m). The distances between plants and rows were 25 and 75 cm, respectively.

### 2.2. Soil Analysis and Climatic Conditions

Prior to the sowing date, soil samples were collected from three different locations with an auger (2.0 cm in diameter) for physical and chemical analysis of the soil (Table 1 and Table 2), as described in the methods of soil analysis by Page et al. [22] and Klute [23]. The soil samples were well mixed prior to analysis for the accuracy of the results. The soil was a clay loam with clay 36.39%, silt 22.88%, fine sand 36.40%, coarse sand 4.33%, pH 7.49, EC 2.44 dS m^−1^, available N 51.0 mg kg^−1^, available K 400.0 mg kg^−1^, available P 22.30 mg kg^−1^, field capacity 35.8%, wilting point 18.8%, available water 17.0%, and bulk density 1.21 Mg m^−3^.

The climate variables in the investigated area were averaged for each month (Table 3). The total precipitation during the crop season (from October to January) was 77.6 mm per growing season. The average maximum and minimum air temperature was 24.2 and 12.6 °C, respectively. In addition, the average relative humidity was 60.0%.

### 2.3. Fertilization

Fertilization of potato in terms of the amount and the method of application were done according to the recommendation of the Egyptian Ministry of Agriculture in the investigated area. Nitrogen was applied in four doses with the total amount of 380 kg N ha^−1^ and the source of N in each plot depending on the treatment of N source (presented in second paragraph of material and methods). The first dose of N was 10% of the total N application and was added during the seed-bed preparation. The second, third, and fourth doses of N were 30%, 30%, and 30% of the total N application, respectively, and were added every 20 days. Potassium (K_2_O) was applied to all treatments at the rate of 192 kg K ha^−1^ in three equal doses. The first dose was added during the seed-bed preparation, while the remaining splits were added every 18 days thereafter. Both N and K fertilizers were banded under the dripper’s line. Phosphorus was applied at the rate of 40 kg P ha^−1^ as ordinary calcium super phosphate (68 g P kg^−1^) during seed-bed preparation. All other agronomic practices were performed in line with the farmers in the area.

### 2.4. Measurements

#### 2.4.1. Crop-Soil-Water Relations

##### Reference Crop Evapotranspiration (ET_o_)

ET_o_ values were calculated based on local meteorological data of the experimental site (Table 3) and according to Equation (1), which is described by Penman–Monteith [24]. Calculations were performed using the CROPWAT model [25] as follows:

(1) $${\mathrm{ET}}_{o}=\frac{0.408∆\left(R_{n}-G\right)+\gamma \;\frac{900}{T+273}U_{2}\left(e_{s}-e_{a}\right)}{∆+\;\gamma \;\left(1+0.34U_{2}\right)}$$

where:

ET_o_: reference evapotranspiration (mm day^−1^),
R_n_: net radiation at the crop surface (MJ m^−2^ day^−1^),
G: soil heat flux density (MJ m^−2^ day^−1^),
T: mean daily air temperature at 2 m height (°C),
u_2_: wind speed at 2 m height (m s^−1^),
e_s_: saturation vapor pressure (kPa),
e_a_: actual vapor pressure (kP),
e_s_ − e_a_: vapor pressure deficit (kPa),
Δ: slope of the vapor pressure-temperature curve (kPa °C^−1^),
γ: psychrometric constant (kPa °C^−1^).

##### Crop Evapotranspiration (ETc)

The ETc values were calculated according to the following, Equation (2) stated by Food and Agricultural Organization (FAO) [26]:

(2) $$\mathrm{ETc}={\mathrm{ET}}_{o}\;\times \;\mathrm{Kc}$$

where:

ETc: crop evapotranspiration (mm day^−1^),
ET_o_: reference crop evapotranspiration (mm day^−1^),
Kc: crop coefficient.

##### Applied Irrigation Water (AIW)

The amounts of applied irrigation water (Table 4) were calculated according to Equation (3) stated by Vermeiren and Jopling [27]:

(3) $$\mathrm{AIW}=\frac{\mathrm{ETc}\;\times \;I}{\mathrm{Ea}\;\left(1-\mathrm{LR}\right)}$$

where:

AIW: depth of applied irrigation water (mm),
ETc: crop evapotranspiration (mm day^−1^),
I: irrigation interval (days),
Ea: irrigation application efficiency for the drip irrigation system (≈ 85% at the site),
LR: leaching requirements: The extra amount of applied water needed for salt leaching, calculated according to FAO [28] as follows:

(4) $$\mathrm{LR}=\frac{\mathrm{ECiw}}{\mathrm{ECe}}$$

where:

ECiw: salinity of irrigation water (dS m^−1^),
ECe: average of soil salinity tolerated by the crop, as measured by soil-saturated extract (dS m^−1^). Under the current experimental conditions, no additional water was added for leaching to avoid any effect on the stress treatments.

##### Water Consumptive Use (WCU)

Water consumptive use (WCU) was determined using a time domain reflectometry (TDR) sensor, which measures the volumetric soil moisture at 0.6 m soil depth before and after each irrigation. The TDR is widely used to measure soil water content, as described by Dasberg and Dalton [29]. The WCU value was calculated, as described by Israelsen and Hansen [30], using Equation (5):

(5) $$\mathrm{WCU}=\overset{i=4}{\underset{i=1}{\sum }}\frac{\theta 2-\theta 1}{100}\times d$$

where:

WCU: water consumptive use or crop evapotranspiration (mm),
i: number of soil layers,
θ2: soil moisture content after irrigation (% volume basis),
θ1: soil moisture content immediately before irrigation (% volume basis),
d: depth of soil layer (mm).

##### Water Utilization Efficiency (WU_t_E)

Water utilization efficiency (WU_t_E) describes the efficiency of the water applied in yield production. It was determined, as described by Jensen [31], as follows:

(6) $${\mathrm{WU}}_{t}E\;\left({\mathrm{kg}\;m}^{-3}\right)=\frac{\mathrm{Tuber}\;\mathrm{yield}\;\left({\mathrm{kg}\;\mathrm{ha}}^{-1}\right)}{\mathrm{Seasonal}\;\mathrm{AIW}\;\left(m^{3}{\mathrm{ha}}^{-1}\right)}$$

#### 2.4.2. Vegetative Growth, Foliage and Tuber Yields

##### Chlorophyll Content

The chlorophyll content in leaves was analyzed at the BBCH stage 40 [32] (80 days after sowing), following the method described by Costache et al. [33]. The extraction of pigments was performed in stoppered tubes. Samples of potato leaves were prepared with a laboratory homogenizer using almost one gram of fresh leaves. Samples were extracted using 100% acetone with a ratio of 1:50. Homogenized mixture was separated using the centrifugation process for 10 min at 3000 rpm. Then, the measurements were performed using α spectrophotometer at the wavelengths of 645 and 662 nm for chlorophyll a and b determination.

(7) Chlorophyll a = 11.75 A662 − 2.350 A645

(8) Chlorophyll b = 18.61 A645 − 3.960 A662

##### Tuber and Foliage Yields

Samples of nine plants were collected randomly from the middle of each sub-plot at harvest (114 days after planting), when plants were supposed to be ripe for measuring tuber yield and the fresh and dry weight of the above-ground plant parts (foliage yield). The plants were removed from the soil using a fork to recover all the tubers. Then, tubers and foliage were immediately placed in different plastic bags with specified numbers, and fresh weights were directly recorded. Then, plant samples were oven dried at +70 °C for 80 h or until the constant weights were achieved for determining the dry weight of yields, as well as for further analysis.

#### 2.4.3. Chemical Quality Analysis of Tubers

Chemical quality analyses were determined based on dry matter, total protein, and N, P, K, and S contents. The tuber samples (DM) were digested using a concentrated sulfuric and perchloric acids mixture, as described by Chapman and Pratt [34], and then N was determined by the Kjeldahl method. Thereafter, the extract was used to measure the total contents of K, and P by using a flame photometer (Corning 410) and Inductively Coupled Plasma (ICP-JY ULTIMA 2) instruments, respectively. For S analysis, the tubers samples were digested using concentrated nitric acid and perchloric acids mixture as describe by Motsara and Roy [35]. Then a turbidimetric method was used for measuring total content of S using Spectrophotometer at 440 nm (Jenway 6705 UV/Vis) [35]. The total protein content was calculated by multiplying the nitrogen % in tuber with the conversion factor of 6.25 [36].

### 2.5. Statistical Analysis

Data obtained from the integrative effects of different drip irrigation regimes and nitrogen source on water traits, growth, yield, and quality of potato were subjected to the analysis of variance (ANOVA) with the general linear model using PASW statistics 21.0 (IBM Inc., Chicago, IL, USA). The means were compared using Tukey’s multiple range test (at significant difference of *p* ≤ 0.05). In addition, the least significant differences (LSD) and standard error of means (SEM) were also obtained from the data provided by the PASW program.

## 3. Results and Discussion

The results of the current study, under the specific conditions of these field experiments, clearly demonstrate that there were high and significant effects of different drip irrigation regimes and nitrogen source on the total chlorophyll content, tuber yield, foliage yield, WUE, and quality traits of potato.

### 3.1. Water Consumptive Use (WCU, m^3^ ha^−1^)

**Figure 1:**
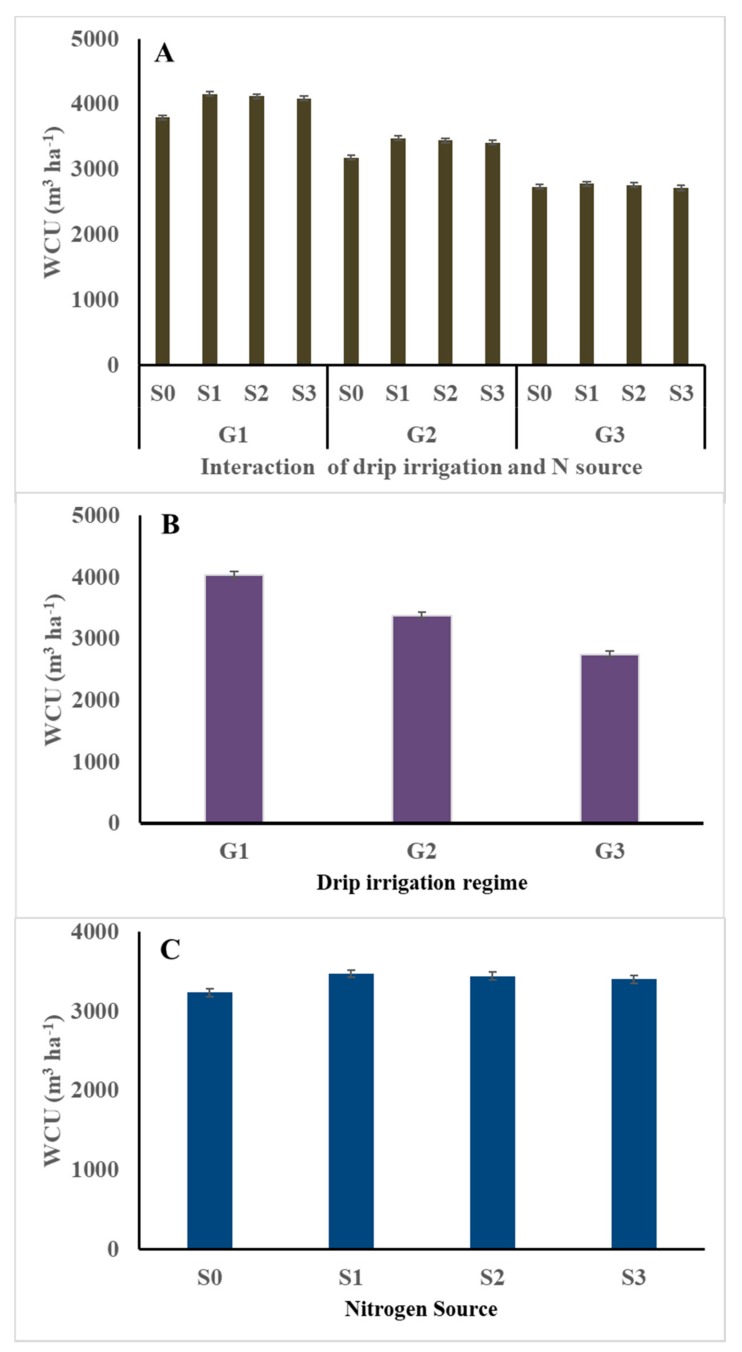
Average effect of drip irrigation regime (**B**), and its interaction with N source (**A**) on water consumptive use (WCU; m^3^ ha^−1^) by potato (average of the two seasons). G_1_ = 120% ETc irrigation; G_2_ = 100% ETc irrigation; G_3_ = 80% ETc irrigation; S_0_ = Unfertilized (control); S_1_ = Urea; S_2_ = Ammonium nitrate; S_3_ = Ammonium sulfate; and Error bars = Standard error of means (SEM).

Water consumptive use (WCU) was the lowest (2732 m^3^ ha^−1^) in potato plants grown with G_3_S_0_ treatment (80% ETc irrigation and non-N fertilizer), while it was the highest in potatoes grown with G_1_S_1_ (4156 m^3^ ha^−1^), G_1_S_2_ (4120 m^3^ ha^−1^), and/or G_1_S_3_ (4085 m^3^ ha^−1^) treatments (highest drip irrigation regime and any N-source) (Figure 1). Low WCU reflects the absence of N fertilization combined with the low amount of water given to the crop. The main effects of nitrogen source treatments followed the pattern S_1_ > S_2_ > S_3_ > S_0_, indicating the lowest WCU by the non-fertilized plants and the highest WCU by the urea-fertilized plants. The main effects of drip irrigation regime demonstrated the following: G_1_ (high) > G_2_ (medium) > G_3_ (low). High soil moisture content in plots irrigated with G_1_ might provide the highest levels of water availability for crop growth, and consequently the highest WCU (Figure 1). Conversely, low moisture content in plots irrigated with the lowest drip irrigation treatment (G_3_) can result in the highest reduction in the WCU_._ The results obtained in the current investigation are consistent with those reported by Eid et al. [37], who stated that increasing the application of irrigation water permits the consumption of water as needed.

**Figure 2:**
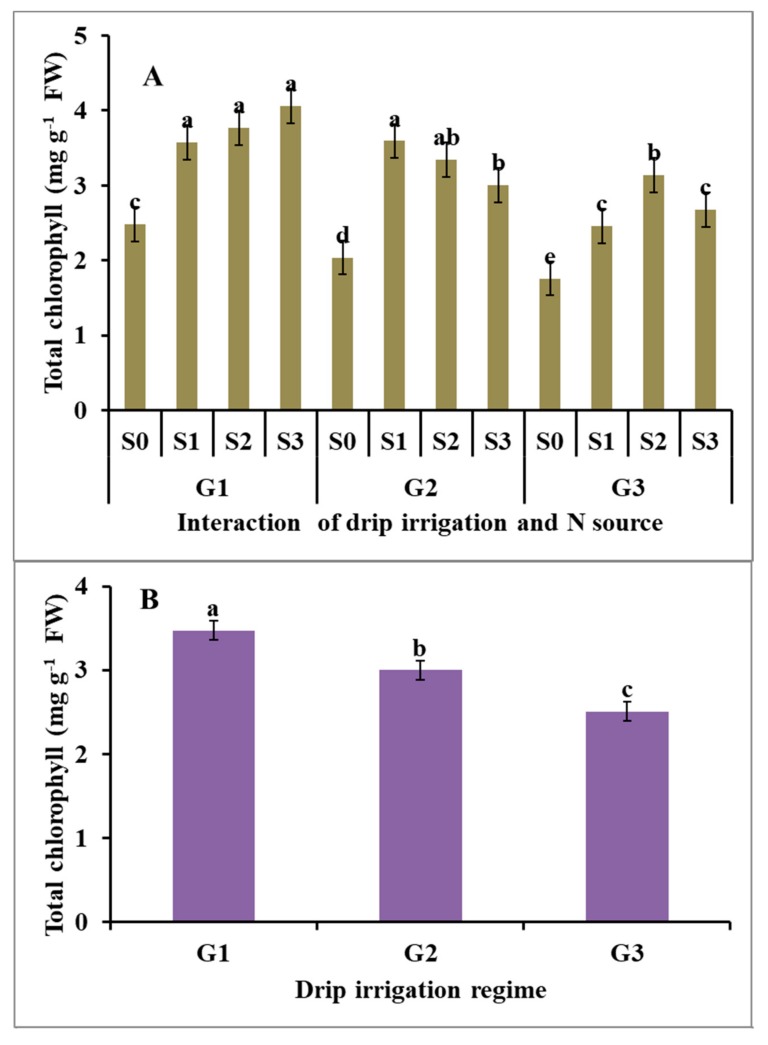

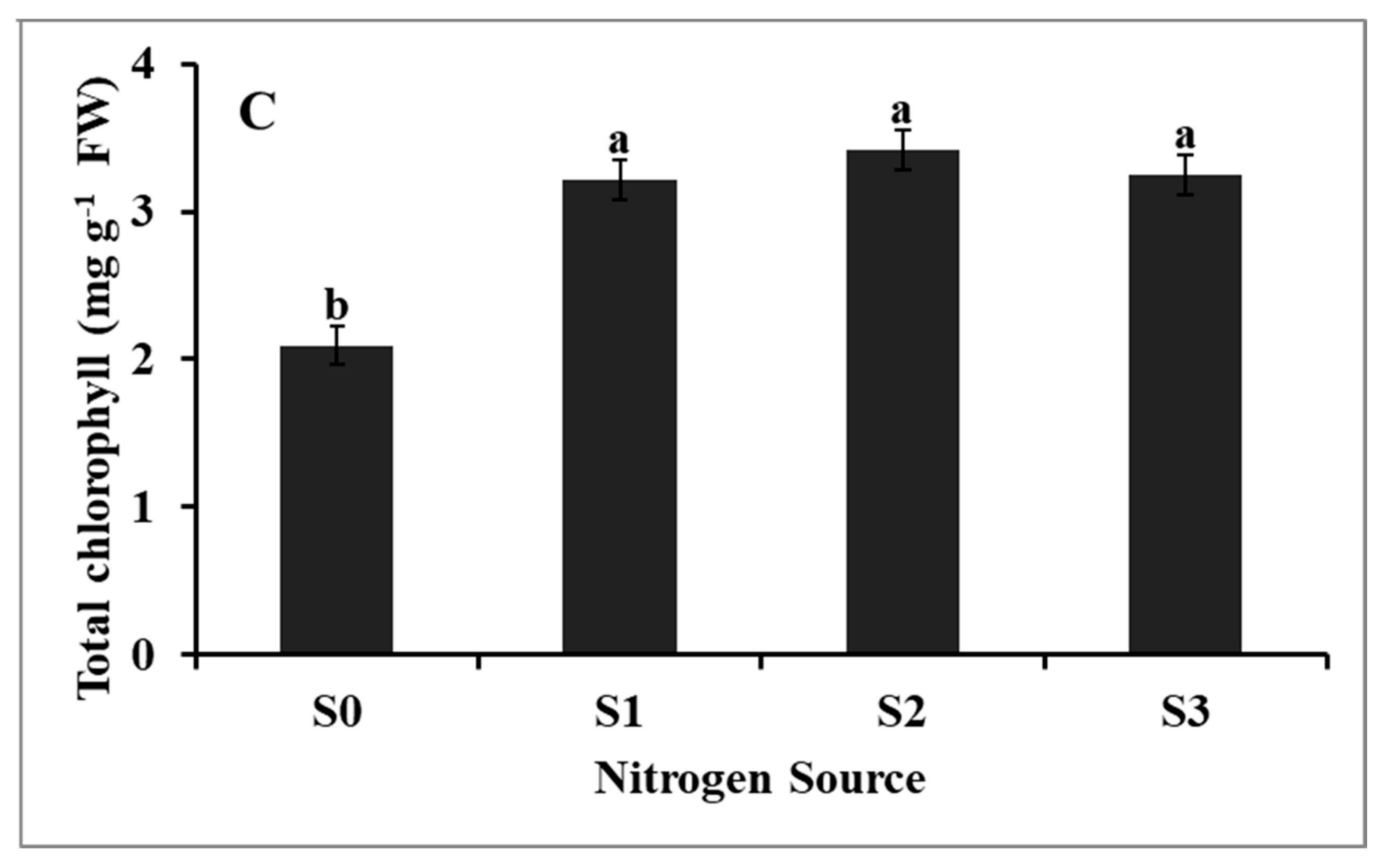
Average effect of drip irrigation regime (**B**), N source (**C**) and their interaction (**A**) on the total chlorophyll (mg g^−1^ FW) of potato. G_1_ = 120% ETc irrigation; G_2_ = 100% ETc irrigation; G_3_ = 80% ETc irrigation; S_0_ = Unfertilized (control); S_1_ = Urea; S_2_ = Ammonium nitrate; S_3_ = Ammonium sulfate; and Error bars = Standard error of means (SEM). Values for the same parameter without a common letter were significantly differed (*P* ˃ 0.05).

Integrative effects of drip irrigation regime and N source on total chlorophyll content of potato reduced the drip irrigation regime from 120% ETc to 100 and 80% ETc significantly and reduced the total chlorophyll content in potato plants by 12% and 28%, respectively (Figure 2). While there were no significant differences among the effects of the three nitrogen source on the total chlorophyll content of potato plants, these nitrogen sources resulted in higher total chlorophyll content compared with unfertilized potato plants (Figure 2). Concerning the interaction effect, the application of different nitrogen sources minimized the negative impact of drip irrigation reduction on total chlorophyll content. Under the highest drip irrigation regime (i.e., 120% ETc), ammonium sulfate, ammonium nitrate, and urea significantly increased the total chlorophyll content by 64%, 52%, and 44%, compared to unfertilized plants, respectively (Figure 2). However, the application of urea, ammonium nitrate, and ammonium sulfate significantly increased the total chlorophyll content by 76%, 64%, and 47%, compared to unfertilized potatoes irrigated with the medium drip irrigation regime (i.e., 100% ETc), respectively (Figure 2). The potato is a crop sensitive to water deficit [11]. Slight water stress can cause a reduction in chlorophyll content and photosynthesis; accordingly, this can affect the size of the marketable tubers [11,38]. In addition, the supply of nitrogen in the proper form can stimulate root growth and the development of potatoes, as well as the uptake of the essential nutrients [39] because it is an essential constituent of protein and chlorophyll [40].

### 3.2. Yield of Potato Tubers

Potato tuber yield varied with the application of different drip irrigation regime and nitrogen source (Table 5). The lowest tuber yield of 10.15 Mg ha^−1^ was obtained from the lowest drip irrigation regime (G_3_; 80% ETc) under non-fertilized conditions (S_0_). Under non-N fertilization conditions, drip irrigation regime of G_2_ (100% ETc) and G_1_ (120% ETc) increased the tuber yield by 71.6% and 142.8%, respectively, compared with G_3_ (Table 5). This indicates that high irrigation increased the yield of tubers, and severe water stress could have significantly reduced the tuber yield. Under different drip irrigation regime, different nitrogen sources in the current investigation resulted in higher tuber yields than that obtained from non-fertilized plants. For instance, the application of G_3_S_1_ and G_1_S_2_ increased the tuber yield by 409% and 548%, in comparison to G_3_S_0_, respectively.

However, the results showed that there was no significant difference between the effect of ammonium nitrate and sulfate on the tuber yield of potato under drip irrigation regime of G_1_ and G_2_, while the difference was significant under the drip irrigation regime of G_3_ (Table 5). The highest yield obtained from ammonium nitrate could be due to the direct uptake of plants to the nitrate. However, nitrogen applied in the form of ammonium sulfate should first be converted into the nitrate form via the soil microorganisms during the growing season of potato through the nitrification process. Fertilization containing sulfur improved potato yield and tuber quality in terms of protein, starch, carotene, and macro and microelements [41]. Nitrogen form or source application can pay a vital role in inducing nitrogen cycling, crop productivity, nitrogen loss patterns, and nitrogen recovery [42]. The application of nitrogen fertilizer in the NO_3_^−^–form is more susceptible to the leaching and denitrification process, while the application of nitrogen fertilizer in the NH_4_^+^–form is more susceptible to volatilization [42,43]. However, both ammonium nitrate and ammonium sulfate are considered to be the two most common sources used as conventional nitrogen fertilizers in potato production. Different studies have compared the effect of different conventional nitrogen sources on potato yield and quality, with contrasting results. Certainly, the performance of NO_3_^−^ and NH_4_^+^ containing nitrogen fertilizer sources may not be consistent long term, due to the changes in soil and climatic conditions, that is, mainly soil organic matter content and soil pH [44].

**Figure 3:**
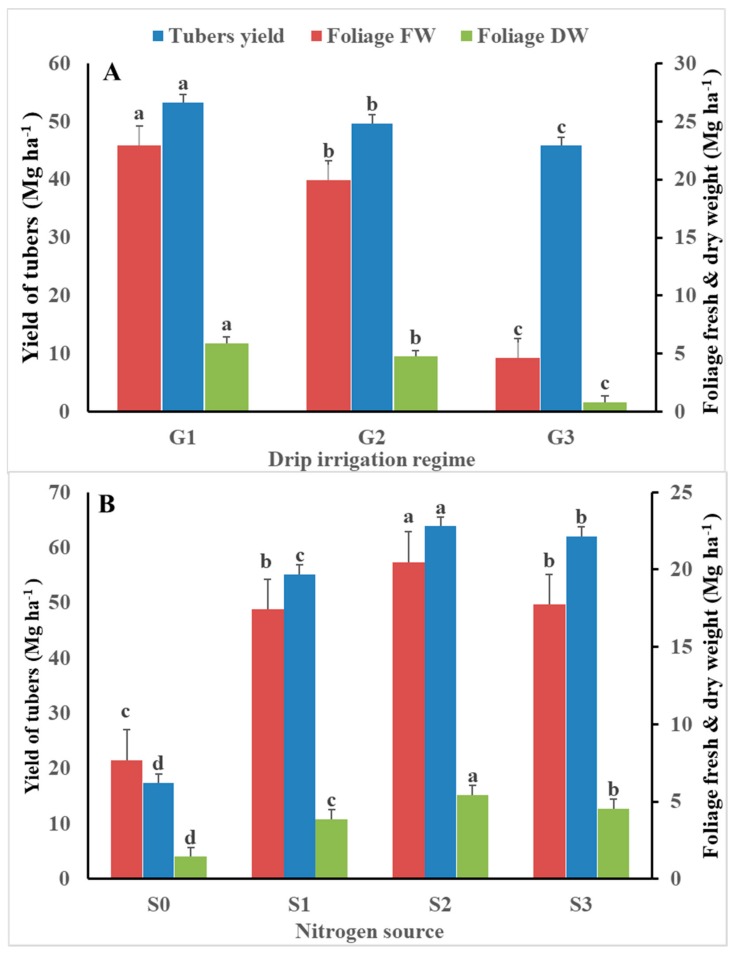
Average effect of drip irrigation regime (**A**) and N source (**B**) on tubers and foliage yields of potato (Mg ha^−1^). FW = fresh weight; DW = dry weight; G_1_ = 120% ETc irrigation; G_2_ = 100% ETc irrigation; G_3_ = 80% ETc irrigation; S_0_ = Unfertilized (control); S_1_ = Urea; S_2_ = Ammonium nitrate; S_3_ = Ammonium sulfate; values for the same parameter without a common letter are significantly differed (*P* ˃ 0.05).

Regarding the response of the drip irrigation regime on tuber yield, the main effect followed the pattern G_1_ > G_2_ > G_3_, with a significant increase of 8.3% and 16.1% for G_2_ and G_1_, respectively, compared with G_3_ (Figure 3a). Drip irrigation regime of 120% and 100% ETc might enhance the regular growth of the shoots, stolon formation, tuber initiation, early stage of tuber bulking, and tuber ripening, which are considered to be sensitive stages to water deficit stress in potatoes [8]. The current results are consistent with those obtained by Khalil [16]. The increasing tuber yield by the increasing irrigation (Figure 3a) might be due to the increase of total chlorophyll content (Figure 2). In addition, it might be due to the increment of leaf transpiration, which is correlated with the increasing water supply [45]. Consequently, this might have a positive effect on yield via the enhancing gases exchange and photosynthesis process [45]. Thus, to achieve high yielding of tubers, the soil water content should be no lower than 50% of the maximum available water in the root zone, particularly during tuber formation [46]. However, Cantore et al. [47] obtained 44.9 and 34.3 Mg tubers ha^−1^ when full irrigation and irrigation at 50% depletion were applied, respectively, compared with non-complementary irrigation (16.5 Mg tubers ha^−1^). Ierna and Mauromicale [8] reported that the highest tuber yield was obtained from plants irrigated with 100% maximum evapotranspiration (ETm). They also reported that the highest irrigation water productive and high tuber quality were obtained from potatoes irrigated with 100% ETm supplied from tuber initiation until 50% tuber growth. Moreover, Ierna and Mauromicale [8] found that this could save a large amount of irrigation water (approximately 77 mm year^−1^), which is considered to be a significant reduction for the semi-arid regions.

The effect of N source treatments on the tuber yield of potato followed the pattern S_2_ > S_3_ > S_1_ > S_0_ (Figure 3b). Compared with non-fertilized plants, ammonium nitrate, ammonium sulfate, and urea significantly increased the tuber yield by 266%, 256%, and 217%, respectively. Marouani et al. [48] reported that there were no significant differences between urea and ammonium nitrate in terms of the total tubers yield of potato. However, Davis et al. [49] suggested that the use of N fertilizer, as NO_3_^−^N or NH_4_^+^NO_3_ instead of NH_4_^+^, resulted in high increases in potato growth and tuber yield. In addition, Wang et al. [50] reported that the nitrate nitrogen content was significantly correlated with the availability of K and P in the soil; consequently, the tuber yield of potato was increased. Gao et al. [17] and Qiqige et al. [51] found that NO_3_^−^-N achieved higher tuber yield compared with NH_4_^+^-N. The current results of our investigation are consistent with those obtained by Weixing and Tibbitts [52] who stated that potato fertilized with a mixture of NH_4_^+^ and NO_3_^−^ improved the potato yield by enhancing nitrogen uptake.

The results of the current investigation are in agreement with those obtained by Bélanger et al. [13], who reported that tuber yield increased when the amount of irrigation water was increased under the drip irrigation system. Additionally, Farrag et al. [53] reported that increasing irrigation water into potato from 50% to 100% ETc caused a significant increase in tuber yield. In addition, Badr et al. [20] stated that the full irrigation of potato resulted in the highest tuber yield with different investigated N levels used in their study, while a significant decrease in the tubers yield occurred in plants irrigated with low amounts of irrigation water. Moreover, El-Mokh et al. [6] reported that irrigation with 100% ETc + 300 kg N ha^−1^ resulted in the highest potato tuber yield. However, Yuan et al. [10] and Onder et al. [54] reported a significant reduction in the tuber yield with decreasing levels of applied water. Alva et al. [21] noted that the reduction in the total applied water from 14% to 20% of full irrigation reduced the total tuber yield from 7% to 28%, compared with full irrigation.

### 3.3. Yield of Fresh and Dry Foliage

Fresh and dry foliage yield of potato were significantly varied with the application of different drip irrigation regimes and nitrogen source treatments (Table 5). The lowest fresh yield (3.52 Mg ha^−1^) was obtained from non-fertilized potatoes under G_3_ drip irrigation (80% ETc). Under non-fertilized treatment, irrigation potatoes with the highest drip irrigation (120% ETc) and the medium irrigation (100% ETc) significantly increased the fresh yield by 235.8% and 119.0%, compared with the lowest drip irrigation regime (80% ETc), respectively. All different nitrogen source treatments resulted in greater fresh foliage yields than the unfertilized plants. The effect of nitrogen source treatments followed the pattern S_2_ > S_3_ > S_1_ > S_0_, indicating that ammonium nitrate followed by ammonium sulfate resulted in the highest fresh and dry foliage yield of potato, while urea application resulted in the lowest yield (Figure 3b). The fertilizers provided average increases of 167%, 131%, and 127% with S2, S3, and S1 compared with unfertilized plants, respectively. The increment in the fresh and dry yield of potato under different nitrogen source applications could be attributed to the positive enhancement of nitrogen on plant growth, photosynthesis and stolons formation by influencing the biosynthesis and activity of phytohormons balance [55].

The effect of drip irrigation followed the pattern G_1_ > G_2_ > G_3_, with average increases of 330% and 395% for G_2_ and G_1_, compared with G_3_, respectively. These results are consistent with those obtained by Ahmed et al. [15] who reported that ammonium nitrate resulted in the highest yield of foliage, followed by the application of urea and ammonium sulfate. However, Marouani et al. [48] reported a significant difference between the application of urea and ammonium nitrate in terms of vegetative growth at the tuber maturation stage. The high vegetative growth obtained using ammonium nitrate, compared with the other sources, indicates that potato plants prefer NO_3_^−^-N over NH_4_^+^-N for high yields [15]. Weixing and Tibbitts [52] stated that potato fertilized with a mixture of NH_4_^+^ and NO_3_^−^ prompted vegetation growth, compared with NH_4_^+^ or NO_3_^−^ alone. However, the application of NH_4_^+^-N resulted in a larger leaf area than NO_3_^−^-N [50]. Henceforth, the overall greater potato growth and productivity with ammonium nitrate than ammonium sulfate may be due, in part, to the presence of both forms on N supplied by ammonium nitrate. Our results might also be due to the slow or delayed nitrification of ammonium sulfate [43]. This was proved when ammonium sulfate resulted in the proper quality traits without significant differences with the ammonium nitrate.

The response pattern observed for foliage dry weight in the current investigation was consistent with that of the fresh weight (Table 5). The lowest dry foliage (0.40 Mg ha^−1^) was obtained from unfertilized plants with the lowest drip irrigation (G_3_; 80% ETc). This yield was surpassed by other, different treatments, with increases of 117.5% with G_3_S_3_ (80% ETc and ammonium sulfate) and up to 20-fold with G_1_S_2_ (120% ETc and ammonium nitrate). Application of S2, S3, and S1 resulted in a significant increase in dry foliage yield by 278%, 217%, and 168% compared with unfertilized plants, respectively (Figure 3). Such increases were more pronounced than those obtained with the fresh foliage yields. Conversely, the main effect for irrigation followed a similar pattern to that of the fresh foliage (i.e., G_1_ > G_2_ > G_3_) with more pronounced average increases than with fresh foliage, which had increases of 619.5% and 479.3% for G_1_ and G_2_, compared to G_3_, respectively. Cantore et al. [47] reported that potato foliage yields were 159, 134, and 70 g m^−2^ with full irrigation, 50% full irrigation, and rain-fed irrigation, respectively.

### 3.4. Water Utilization Efficiency (WUtE)

**Figure 4:**
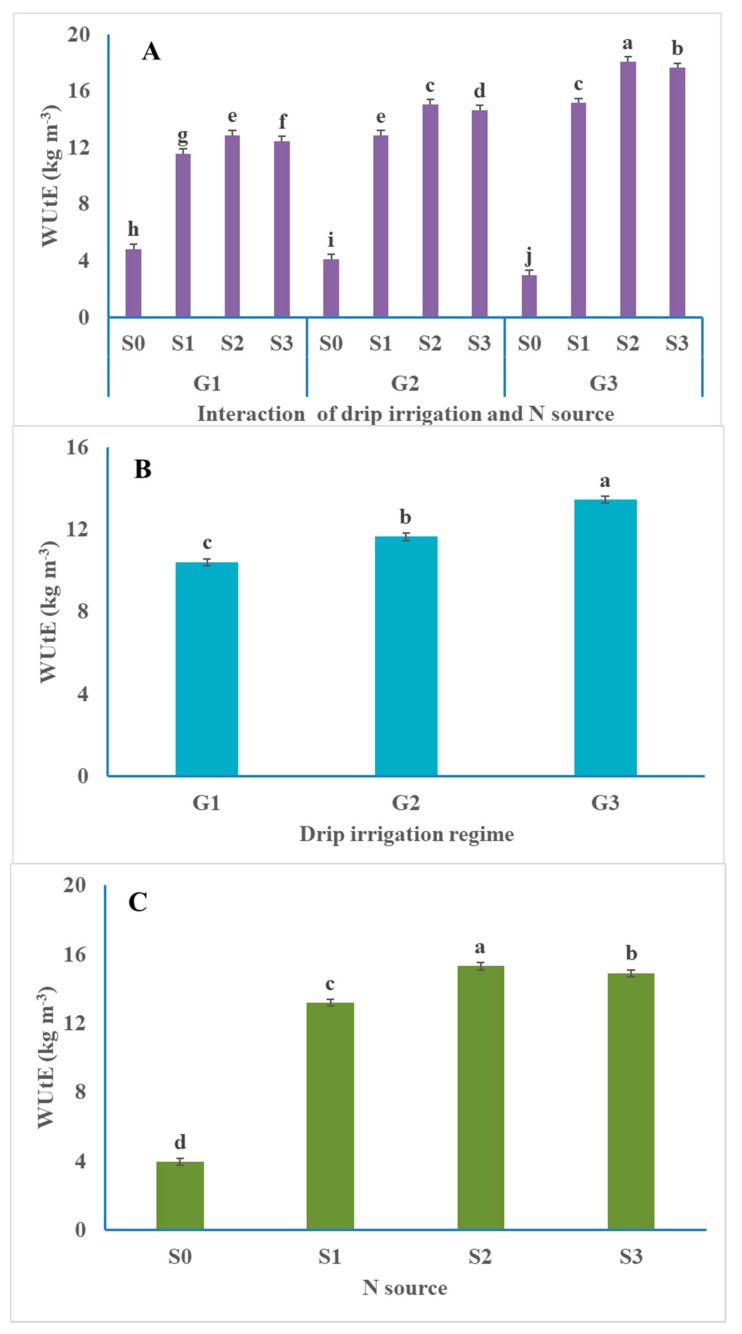
Average effect of drip irrigation regime (**B**), N source (**C**) and their interaction (**A**) on water utilization efficiency (WUtE; kg m^−3^) of potato. G_1_ = 120% ETc irrigation; G_2_ = 100% ETc irrigation; G_3_ = 80% ETc irrigation; S_0_ = Unfertilized (control); S_1_ = Urea; S_2_ = Ammonium nitrate; S_3_ = Ammonium sulfate; values for the same parameter without a common letter are significantly differed (*P* ˃ 0.05).

The WUtE (kg tubers per m^−3^ of applied water) varied with different treatments (Figure 4). All treatments receiving urea, ammonium nitrate, or ammonium sulfate resulted in greater WUtE than those unfertilized with N. The lowest value, 2.98 kg m^−3^, was observed with G_3_S_0_ (unfertilized with N and low irrigation) treatment, while the highest value, 18.05 kg m^−3^ (an increase of 505.7%), was observed with the G_3_S_2_ (low irrigation ammonium nitrate) treatment. The main effect of N treatments followed the pattern S_2_ > S_3_ > S_1_ > S_0_. Unfertilized plants with N had the lowest WUtE, while fertilized plants with different sources of N had the highest WUtE. The urea source was the least efficient, while the ammonium nitrate source was the most efficient for this variable. The superiority of ammonium nitrate and ammonium sulfate over urea was more apparent when medium or low irrigation was used.

The main effect of irrigation followed the pattern G_3_ > G_2_> G_1_; such a pattern occurred following the application of fertilizer N, reflecting the highest WUtE of irrigation treatment achieved where irrigation was given in low amounts (80% ETc). The water utilization efficiency increases of 29.2% and 11.9% for G_3_ and G_2_ over G_1_. Conversely, where no-N fertilizer was applied, the pattern was G_1_ > G_2_ > G_3_, indicating that plant roots may have expanded exploration, receiving high irrigation in order to acquire N under conditions of no-N addition. Cantore et al. [47] noted that water use efficiency was not affected by the applied supplementary irrigation, and reported values of 10.7 and 10.6 for full irrigation and 50% irrigation, respectively, compared with 9.0 kg m^−3^ for rain-fed irrigation. Ritchie [56] noted that medium water stress could increase water conservation. When roots are subjected to soil moisture stress, soil water is extracted from greater depths to allow for the use of water stored in soil. Gültekin and Ertek [57] stated that irrigation, once near-field capacity was reached, achieved the highest tuber yield compared with irrigation upon 60% and 75% depletion of the available water. Cantore et al. [47] stated that the marketable potato tuber yield decreased by 26% and 64% with 50% of full irrigation and when rain-fed, respectively, compared with full irrigation.

Farrag et al. [53] obtained the highest water utilization efficiency at 75% of the water requirements. Iernaa and Mauromicale [8] applied deficient irrigation and increased water productivity. El-Mokh et al. [6] obtained the highest yield under supplementary irrigation, while Bélanger et al. [13] found that irrigation increased the weight per tuber.

### 3.5. Quality of Potato Tubers in Terms of Elemental Analysis and Protein

The results showed that drip irrigation regime and nitrogen source resulted in significant effects on different quality traits of potato tubers in terms of elemental analysis and total protein (Table 6 and Table 7). In addition, the results showed that potato tubers contained the most potassium (range of 16.87−32.78 g kg^−1^), slightly less nitrogen (2.75–11.37 g kg^−1^), considerably less phosphorus (1.97–2.76 g kg^−1^), and sulfur (0.04–1.49 g kg^−1^). This was mainly due to the availability of these elements in the soil of the investigated area.

#### 3.5.1. Nitrogen, Sulfur, and Protein Contents in Tubers

Nitrogen is considered to be essential for different physiological traits and processes in plant cells and accordingly for plant growth and yields; however, some quality traits can be affected adversely by nitrogen fertilization [58]. The highest contents of nitrogen (11.37 g kg^−1^) and total protein (71.06 g kg^−1^) in tubers were obtained when potato plants were grown with G_3_S_3_ (80% ETc with ammonium sulfate), followed by G_3_S_2_, G_2_S_2_, and G_2_S_3_ in comparison to other treatments (Table 6). However, the lowest nitrogen and total protein content were obtained from unfertilized plants with different drip irrigation regimes. The increment of nitrogen and total nitrogen obtained by the application of G_3_S_3_ was over three-fold, compared with those obtained from the application of G_1_S_0_ (Table 6). The link between the protein biosynthesis and the supply of crops with sulfur was identified by many authors [59,60]. Sulfur enhances the activity of the enzymes, which is involved in nitrate reduction [61]. Additionally, it is considered a component of some amino acids such as methionine, cysteine, and cystine, and is consequently indispensable for the biosynthesis of proteins [61].

However, the highest significant content of sulfur was only obtained from plants grown with G_3_S_3_ (1.49 g kg^−1^) and G_2_S_3_ (1.41 g kg^−1^), while the lowest significant content of sulfur was obtained from unfertilized plants under the condition of G_1_ (0.04 g kg^−1^). Our results indicated that ammonium sulfate could provide a significant beneficial impact on the tuber quality due to its nitrogen ratio; moreover, it also provides the added benefit of supplying approximately 24% sulfur, which is considered to be a main component of different essential amino acids that are involved in protein synthesis. After nitrogen, phosphorus, potassium, and magnesium, sulfur plays a vital role in plant metabolism.

According to the individual factors of nitrogen source, the main effect of nitrogen source treatments followed the pattern S_2_ ≥ S_3_ > S_1_ > S_0_, indicating that ammonium nitrate and/or ammonium sulfate resulted in the highest tuber nitrogen and total protein content, while urea resulted in the lowest content (Table 7). In addition, the main effect of drip irrigation regime showed that G_3_ ≥ G_2_ > G_1_, indicating that the lowest and/or the medium drip irrigation regime resulted in the highest nitrogen and total protein contents, while the highest drip irrigation regime resulted in the lowest contents of those traits. In this respect, Ahmed et al. [15] reported that nitrogen application increased nitrogen content in potato tubers, but without significant differences among the investigated three sources of nitrogen. However, Weixing and Tibbitts [52] reported that potato fertilized with a mixture of NH_4_^+^ and NO_3_^−^ contained greater nitrogen content of whole plants compared with NH_4_^+^ or NO_3_^−^ alone.

##### Phosphorus Content in Tubers

The interaction effect on phosphorus content was not significantly varied among most of the nitrogen source with different drip irrigation regimes (Table 6). The highest phosphorus content (2.76 g kg^−1^) was obtained from plants treated with G_1_S_1_, while the lowest phosphorus content (1.97 g kg^−1^) was obtained from plants treated with G_2_S_0_, representing an increment of 40%. However, the main effect of different nitrogen sources followed the pattern S_2_ ≥ S_1_≥ S_3_ > S_0_, indicating that there was no significant differences among the three sources of nitrogen as an individual factor (Table 7). However, this pattern was particularly evident under different drip irrigation regimes, representing the following pattern G_3_ ≥ G_1_> G_2_. Ahmed et al. [15] reported that phosphorus content in tubers was increased by the application of nitrogen fertilization. High content of phosphorus in tubers can cause an increase in the amylose content and changed thermal and pasting starch properties [62]. In addition, it causes an increase in the dry matter, starch, and proteins contents, but decreases total sugar content [63]. Thus, phosphorus is extremely important in the proper development of potato tubers quality.

#### 3.5.2. Potassium Content in Tubers

Potassium content followed different patterns, compared with other elements in potato tubers (Table 6 and Table 7). In this respect, the main effect of different nitrogen sources had the following pattern S_1_ ≥ S_2_ > S_3_ > S_0_, indicating that the application of ammonium nitrate or urea resulted in higher tuber potassium content than ammonium sulfate and unfertilized plants (Table 7). While, the main effect of different drip irrigation regimes had the following pattern G_1_ > G_2_ > G_3_, indicating that G_1_ resulted in higher tuber K content than G_2_ and G_3_. According to the interaction effect, the lowest potassium content (16.87 g K kg^−1^) was obtained from potatoes treated with G_3_S_0_, while the highest potassium (32.78 g K kg^−1^) was obtained from potatoes treated with G_1_S_2_, representing an increment of 94.3%. Winkelmann [64] reported that the concentration of potassium in tubers with the average of 22–25 g K kg^−1^ DW is considered optimal for high yielding and high quality. In our investigation, potassium content in tubes of potatoes grown under most treatments was equal or higher than the optimal level for better quality. In this respect, similar results were obtained by Ahmed et al. [15], who report that nitrogen applications increased potassium content in tubers, and the highest nitrogen was obtained from plants fertilized with ammonium nitrate compared with ammonium sulfate or urea. High rates of crop phosphorus and potassium may cause residual contents of phosphorus and potassium for the subsequent crops [65,66], which must be considered in practice. On the contrary, Tein et al. [67] stated that potassium content in potato tuber was not affected by nitrogen applications.

## 4. Conclusions

Potato quality, tuber and foliage yields, chlorophyll content, and water productivity were significantly affected by drip irrigation regime and N source. The results of our investigation have demonstrated that it is possible to save irrigation water (854 m^3^ season^−1^, difference between drip irrigation of 120% ETc and 100% ETc), and still maintain high tubers yields of potatoes, suitable tuber quality, and high WUtE. In addition, our investigation demonstrated that the most suitable sources of N were S_1_ (ammonium nitrate) and/or S_3_ (ammonium sulfate) for potato growth, yield and proper quality under the recommended rate of 380 kg N ha^−1^. In conclusion, the integrative effects of G_1_ or G_2_ with S_2_ or S_3_ is recommended for high yields of potato, while integrative effects of G_2_S_2_, G_3_S_2_, and G_3_S_3_ are recommended for high quality tubers with the notification of low tuber yield. The above irrigation water quantity saved for a single potato season means a significant water quantity for the semi-arid regions, and consequently, considerable savings at national and regional scales. Even though the effectiveness of drip irrigation at 100% ETc may raise water savings for irrigated crops such as potato in the short term, plant breeders should work hard to produce efficient genotypes regarding water saving long-term.

## Figures and Tables

**Table 1:** Main soil moisture parameters and soil bulk density of the experiments.

Depth (cm)	Field Capacity (FC)		Wilting Point (WP)		Available Water (AW)		Bulk Density (Bd), Mg m^−3^
	%	cm	%	cm	%	cm	
0–15	35.8	6.50	18.8	3.41	17.0	3.10	1.21
15–30	33.4	5.91	17.3	3.10	16.1	2.81	1.18
30–45	31.9	5.98	15.1	2.83	16.8	3.15	1.25
45–60	31.7	7.23	16.8	3.83	14.9	3.4	1.52

**Table 2:** Main physical and chemical properties of soil in the investigated area.

Parameter	Value
CaCO_3_^−^ (gkg^−1^)	42.90
Organic matter (g kg^−1^)	20.50
Total N (mg kg^−1^)	210.00
Available N (mg kg^−1^)	51.00
Available K (mg kg^−1^)	400.00
Available P (mg kg^−1^)	22.30
pH (1:2.5 *w/v* soil: water suspension)	7.49
EC dS m^−1^ (paste extract)	2.44
Saturation %	53.00
Cations and anions in soil paste extract (m mol_c_ L^−1^)	
Na^+^	3.57
K^+^	0.97
Ca^2+^	17.05
Mg^2+^	2.86
CO_3_^2−^	0.00
HCO_3_^−^	5.42
Cl^−^	9.32
SO_4_^2−^	9.71
Clay (%)	36.39
Silt (%)	22.88
Fine sand (%)	36.40
Coarse sand (%)	4.33
Texture (International Texture Classification)	Light Clay

**Table 3:** Meteorological data of the experimental site, crop coefficient (Kc), and crop evapotranspiration (ETo) in the 2015/16 and 2016/17 seasons (the average of the two seasons).

Month	Temperature (°C)		WS (ms^−1^)	RH (%)	SD (h)	Precipitation (mm Month^−1^)	Kc	ETo (mm Month^−1^)
	(Max.)	(Min.)						
October	31.7	18.3	3.7	55	11.2	17.5	0.50	5.87
November	26.5	14.2	3.6	57	10.4	25.2	0.78	4.45
December	20.8	10.4	4.1	64	10.1	24.7	1.11	3.32
January	17.9	7.8	3.9	64	10.3	10.2	0.67	2.48
Mean	24.2	12.6	3.8	60.0	10.5	19.4	0.76	4.03

WS = Wind speed; RH = Relative humidity; SD = Sunshine duration; Kc = Crop coefficient; ETo = reference crop evapotranspiration.

**Table 4:** Average monthly and seasonal irrigation water supplied to the potato crop.

Month	Irrigation Treatment		
	G_1_	G_2_	G_3_
	m^3^ Month^−1^	m^3^ Month^−1^	m^3^ Month^−1^
October	988	823	659
November	1661	1384	1107
December	1773	1477	1182
January	697	581	464
Total (m^3^ season^−1^)	5119	4265	3412

G_1_ = 120% ETc irrigation; G_2_ = 100% ETc irrigation; G_3_ = 80% ETc irrigation.

**Table 5:** Tubers and foliage yields of potato (Mg ha^−1^) as affected by drip irrigation regime and N source interaction.

Irrigation Regime (G)	N Source (S)	Yield of Tubers (Mg ha^−1^)	Foliage Fresh Weigh (Mg ha^−1^)	Foliage Dry Weigh (Mg ha^−1^)
G_1_	S_0_	24.64 ^e^	11.82 ^c^	2.68 ^d^
	S_1_	59.04 ^b^	25.10^ab^	5.49 ^c^
	S_2_	65.79 ^a^	28.88 ^a^	8.28 ^a^
	S_3_	63.60 ^a^	25.91 ^a^	7.13 ^b^
G_2_	S_0_	17.42 ^f^	7.71 ^d^	1.23 ^e^
	S_1_	54.79 ^c^	22.49 ^ab^	5.20 ^c^
	S_2_	64.11 ^a^	27.07 ^a^	6.91 ^b^
	S_3_	62.40 ^ab^	22.43 ^b^	5.67 ^c^
G_3_	S_0_	10.15 ^g^	3.52 ^e^	0.40 ^e^
	S_1_	51.64 ^d^	4.69 ^d^	0.89 ^e^
	S_2_	61.57 ^ab^	5.48 ^d^	1.13 ^e^
	S_3_	60.11 ^b^	4.84 ^d^	0.87 ^e^

G_1_ = 120% ETc irrigation; G_2_ = 100% ETc irrigation; G_3_ = 80% ETc irrigation; S_0_ = Unfertilized (control); S_1_ = Urea; S_2_ = Ammonium nitrate; S_3_ = Ammonium sulfate; values in the same column without a common letter are significantly differed (*P* ˃ 0.05).

**Table 6:** Chemical composition of potato tubers as affected by drip irrigation regime and N source interaction.

Irrigation Regime (G)	N source (S)	Nitrogen (g kg^−1^)	Phosphorus (g kg^−1^)	Potassium (g kg^−1^)	Sulfur (g kg^−1^)	Protein (g kg^−1^)
G_1_	S_0_	4.24 ^c^	2.10 ^ab^	28.04 ^b^	0.04 ^d^	26.50 ^c^
	S_1_	6.63 ^b^	2.76 ^a^	32.29 ^a^	1.01 ^c^	41.44 ^b^
	S_2_	8.55 ^ab^	2.50 ^a^	32.78 ^a^	1.19 ^b^	53.44 ^ab^
	S_3_	7.95 ^b^	2.38 ^ab^	24.76 ^bc^	1.28 ^b^	49.69 ^b^
G_2_	S_0_	3.39 ^c^	1.97 ^b^	24.46 ^c^	1.04 ^c^	21.19 ^c^
	S_1_	9.60 ^ab^	2.21 ^ab^	25.22 ^bc^	1.10 ^bc^	60.00 ^ab^
	S_2_	10.25 ^a^	2.52 ^a^	22.93 ^c^	1.27 ^b^	64.06 ^a^
	S_3_	9.80 ^ab^	2.28 ^ab^	24.29 ^c^	1.41 ^a^	61.25 ^ab^
G_3_	S_0_	2.75 ^c^	2.60 ^d^	16.87 ^d^	1.14 ^bc^	17.19 ^c^
	S_1_	9.03 ^ab^	2.42 ^ab^	25.66 ^bc^	1.25 ^b^	56.44 ^ab^
	S_2_	10.83 ^a^	2.55 ^a^	26.65 ^b^	1.33 ^ab^	67.69 ^a^
	S_3_	11.37 ^a^	2.50 ^a^	26.29 ^b^	1.49 ^a^	71.06 ^a^

G_1_ = 120% ETc irrigation; G_2_ = 100% ETc irrigation; G_3_ = 80% ETc irrigation; S_0_ = Unfertilized (control); S_1_ = Urea; S_2_ = Ammonium nitrate; S_3_ = Ammonium sulfate; values in the same column without a common letter are significantly differed (*P* ˃ 0.05).

**Table 7:** Main effects of drip irrigation regime and N source on chemical composition of potato tubers (g kg^−1^).

	Parameters	Nitrogen (g kg^−1^)	Phosphorus (g kg^−1^)	Potassium (g kg^−1^)	Sulfur (g kg^−1^)	Protein (g kg^−1^)
Treatments						
Irrigation regime (G)						
G_1_		6.84 ^b^	2.44 ^a^	29.47 ^a^	0.87 ^c^	42.75 ^b^
G_2_		8.26 ^a^	2.25 ^b^	24.23 ^b^	1.20 ^b^	51.63 ^a^
G_3_		8.50 ^a^	2.52 ^a^	23.87 ^c^	1.30 ^a^	53.13 ^a^
N source (S)						
S_0_		3.46 ^c^	2.22 ^b^	23.12 ^c^	0.73 ^d^	21.63 ^c^
S_1_		8.42 ^b^	2.46 ^ab^	27.72 ^a^	1.12 ^c^	52.63 ^b^
S_2_		9.88 ^a^	2.52 ^a^	27.45 ^a^	1.27 ^b^	61.75 ^a^
S_3_		9.71 ^a^	2.39 ^ab^	25.11 ^b^	1.40 ^a^	60.69 ^a^

G_1_ = 120% ETc irrigation; G_2_ = 100% ETc irrigation; G_3_ = 80% ETc irrigation; S_0_ = Unfertilized (control); S_1_ = Urea; S_2_ = Ammonium nitrate; S_3_ = Ammonium sulfate; values in the same column without a common letter are significantly differed (*P* ˃ 0.05).
